# Age-Related Differences in Optical Coherence Tomography and Optical Coherence Tomography Angiography Parameters Between Healthy Children and Adults: A Comparative Analysis in a Caucasian Population

**DOI:** 10.3390/jpm15120629

**Published:** 2025-12-17

**Authors:** Claudia Lommatzsch, Antoine Capucci, Swaantje Grisanti, Carsten Heinz, Kai Rothaus

**Affiliations:** 1 Departmentof Ophthalmology, St. Franziskus-Hospital Münster, Hohenzollernring 74, 48145 Münster, Germany; antoine.capucci@augen-franziskus.de (A.C.); carsten.heinz@augen-franziskus.de (C.H.); kai.rothaus@augen-franziskus.de (K.R.); 2 Department of Ophthalmology, University of Lübeck, 23538 Lübeck, Germany; swaantje.grisanti@uksh.de; 3 Department of Ophthalmology, University of Essen, Hufelandstrasse 55, 45147 Essen, Germany

**Keywords:** optical coherence tomography, OCT angiography, pediatric ophthalmology, retinal nerve fiber layer, macular thickness, retinal vascular density, age comparison, developmental differences

## Abstract

**Purpose**: Current pediatric ophthalmology practice relies on adult reference values for optical coherence tomography (OCT) and optical coherence tomography angiography (OCT-A) interpretation due to limited age-appropriate normative data, potentially leading to diagnostic misclassification. **Methods**: We conducted a prospective, cross-sectional study comparing OCT and OCT-A parameters between 37 healthy Caucasian children (1–17 years) and 28 adults (19–65 years) using identical Zeiss CIRRUS protocols. Parameters included peripapillary retinal nerve fiber layer (RNFL), macular thickness, ganglion cell-inner plexiform layer (GCIPL), optic nerve head (ONH) perfusion, and macular vascular density. **Results**: Children exhibited significantly thinner parafoveal macular thickness compared to adults (251.67 ± 21.32 vs. 270.36 ± 17.02 μm; *p* < 0.001) while RNFL thickness remained comparable. OCT-A demonstrated higher ONH perfusion in children across multiple sectors (*p* < 0.001). Within the pediatric cohort, younger children (1–9 years) showed higher macular vessel and perfusion density than older children (10–17 years). All pediatric scans achieved excellent image quality with no exclusions. **Conclusions**: Clinically significant age-related differences in retinal structure and vasculature necessitate pediatric-specific reference ranges. The demonstrated technical feasibility supports routine OCT/OCT-A implementation in pediatric practice with age-appropriate interpretation guidelines.

## 1. Introduction

The pediatric visual system undergoes prolonged maturation extending into late adolescence, involving both functional and potentially structural developmental changes [1,2]. Various pathologies including optic nerve glaucoma, optic neuritis and glioma can threaten the visual pathway during childhood, making early diagnosis essential for optimal visual development.

Modern ophthalmology benefits from advanced imaging techniques that enable structural visualization of nerve fibers through optical coherence tomography (OCT) as well as ocular perfusion through OCT angiography (OCT-A). In adult patients, device manufacturers provide ophthalmologists with automated comparison of measured healthy values against normative databases, facilitating immediate detection of abnormalities. However, such age-appropriate normative databases are not available for pediatric populations, forcing clinicians to rely on adult reference values when evaluating children. This creates significant diagnostic challenges, as developmental differences may be misinterpreted as pathological findings, or, conversely, early pathological changes may be overlooked.

The existing literature on pediatric OCT and OCT-A parameters is characterized by a notable scarcity of comparative studies examining differences between children and adults, with this limitation being more pronounced for OCT-A than for structural OCT parameters. Among the few studies that have attempted pediatric–adult comparisons, most have employed indirect methodologies, comparing pediatric measurements against pre-existing adult normative databases rather than conducting direct comparative analyses [3,4]. While these indirect approaches provide valuable insights, they introduce potential methodological limitations including device-specific variations and population heterogeneity between comparison groups.

Our study addresses this methodological gap by employing a direct comparative approach, measuring both pediatric and adult populations using identical OCT and OCT-A protocols within a single study framework to characterize differences and highlight the need for pediatric reference ranges in clinical practice. Specifically, the primary objective of this study is to characterize retinal nerve fiber layer thickness, macular thickness, and retinal vascular density parameters in healthy pediatric subjects using tabletop Zeiss Cirrus HD-OCT with AngioPlex technology. Our secondary objectives include evaluating the technical feasibility of tabletop OCT imaging in pediatric populations and identifying age-related differences in retinal architecture that may inform future larger-scale normative studies and clinical decision-making in pediatric ophthalmology practice.

The feasibility of OCT imaging in pediatric populations was initially established through pioneering work using handheld OCT systems. Maldonado et al. demonstrated the first successful retinal imaging in awake infants using handheld spectral-domain OCT, establishing technical protocols that made pediatric retinal imaging clinically viable [3]. The Duke group led by Cynthia Toth has further advanced the field through comprehensive work on pediatric retinal OCT and its connection to brain development, providing foundational understanding of developmental retinal changes [4]. Similarly, the Leicester group led by Gottlob and Proudlock provided crucial early normative data on optic nerve head development in healthy infants and children using handheld spectral-domain OCT, demonstrating age-related changes in children as young as newborns [5]. These foundational studies using portable systems paved the way for the current generation of tabletop OCT devices to be successfully adapted for pediatric use.

## 2. Methods

### 2.1. Study Design and Participants

This prospective, single-center investigation was conducted at the Department of Ophthalmology, St. Franziskus-Hospital Münster (2019–2025) following approval from the Ethics Committee of Westfalen-Lippe (No. 2019-245-f-s) and adherence to the Declaration of Helsinki.

Healthy Caucasian volunteers were enrolled after informed consent; exclusion criteria encompassed inadequate cooperation, media opacities, history of retinal perfusion or macular disease, systemic vascular pathology or vasoactive therapy, prior ocular surgery, history of premature birth, and suboptimal image quality. Only one eye per participant (preferentially right) was analyzed. The children’s group was divided into two subgroups: 1–9 years old and 10–17 years old. Similarly, the adult group was subdivided into younger adults (19–31 years) and older adults (≥32 years).

### 2.2. Study Procedures

All participants underwent comprehensive ophthalmological examination including, refraction, best-corrected visual acuity, slit-lamp microscopy, binocular indirect fundoscopy, and intraocular pressure measurement using iCcare rebound tonometry. Biometry was performed using IOL-Master 700 (Carl Zeiss Meditec AG, Jena, Germany) for axial length, lens thickness, and anterior chamber depth measurements.

1. OCT and OCT-A imaging were performed using Zeiss CIRRUS OCT AngioPlex (software version 11). All scans were performed with undilated pupils due to potential vasoactive effects of mydriatics. Signal strength thresholds were predefined (≥6 for structural ONH and macular cubes; ≥8 for OCT-A) and applied uniformly.
  ◦ Zeiss CIRRUS HD-OCT:
    ￭ Optic nerve head (ONH):
      1. Peripapillary retinal nerve fiber layer (RNFL): Optic Disc Cube 200 × 200
        The RNFL Thickness (RNFLT) is displayed in a circular graph divided into four quadrants (superior, nasal, inferior, temporal) and twelve clock-hour sectors.
    ￭ Macular Cube (512 × 128)
      1. Macula Thickness (MT): Automatic segmentation between inner limiting membrane (ILM) and retinal pigment epithelium (RPE). Measurements displayed in three concentric rings: central (fovea), parafoveal (3 mm), and perifoveal (6 mm), both outer rings divided into four quadrants (superior, nasal, inferior, temporal).
      2. Macula Macular Ganglion Cell–Inner Plexiform Layer (GCIPL): The Combined thickness of ganglion cell layer (GCL) and inner plexiform layer (IPL) within 6 × 6 mm cube. Measurements displayed in elliptical annulus with six segments (nasal inferior, temporal inferior, nasal superior, superior, inferior, temporal superior) centered on fovea.
  ◦ Zeiss AngioPlex OCT-A:
    ￭ ONH-Angiography: The Automatic segmentation within 4.5 × 4.5 mm scan area centered on optic disk at radial peripapillary capillary level. Measurements include perfusion (PF %) and flow index (FI) for complete outer region and four quadrants (superior, nasal, inferior, temporal).
    ￭ Macular-Angiography: Automatic segmentation of superficial capillary plexus measuring vessel density (VD) and perfusion density (PD%) in 3 mm and 6 mm protocols.
      3 × 3 mm scan: Central foveal circle (1 mm), complete area, and parafoveal region complete (3 mm diameter without central foveal circle), subdivided into four quadrants.
      6 × 6 mm scan: Central foveal (1 mm), parafoveal and perifoveal regions (extending to 6 mm), each with quadrant subdivisions.
      Foveal avascular zone (FAZ): Automatically quantified using area (mm^2^), perimeter (mm), and circularity index.
    ￭ OCT-A Parameter Definitions (Zeiss AngioPlex)
      *ONH:*
      - Perfusion (PF %): Ratio of vessel to total area.
      - Flow Index (FI): Intensity of perfusion excluding large vessels, focusing on small capillaries. More sensitive to capillary vessel loss at the optic disk margin.
      *Macula:*
      - Vessel Density (VD): Total length of perfused vessels per unit area (mm/mm^2^). Detects loss of individual capillary vessels independent of vessel size.
      - Perfusion Density (PD %): Percentage area of perfused vessels in a measurement region. Accounts for vessel width in addition to length. Calculated as: (Number of pixels with perfused vessels/Total number of pixels) × 100%.
      - Foveal Avascular Zone (FAZ): Central avascular region defined by capillary network boundaries, quantified automatically using area, perimeter, and circularity index.

### 2.3. Statistical Analysis

Data were analyzed using R statistical software (version 4.5.0). Descriptive statistics were calculated with continuous data presented as mean ± standard deviation. Normal distribution was assessed using the Shapiro–Wilk test. Group differences evaluated by Student’s *t*-test or Mann–Whitney U as appropriate, with significance declared at *p* < 0.05. For group comparisons the effect size is presented by Cohen’s d. Sample size for this study was calculated to detect a medium-sized effect (Cohen’s d = 0.8). With a power of 0.8, this results in at least 26 cases per group to be analyzed.

## 3. Results

### 3.1. Study Population (Table 1)

The study cohort comprised 65 participants: 37 children (mean age 10.20 ± 4.42 years) and 28 adults (mean age 37.78 ± 12.49 years). The pediatric subset included 23 children aged 1–9 years (mean 7.25 ± 1.72) and 14 aged 10–17 years (mean 15.05 ± 2.90). Adults were stratified into younger adults (19–31 years, mean 28.87 ± 2.55 years, n = 14) and older adults (≥32 years, mean 46.70 ± 12.11 years, n = 14). Sex distribution differed non-significantly (children 56.8% female, adults 82.1% female, *p* = 0.058). Biometric differences were consistent with normal growth: children had shorter axial length (22.90 ± 0.99 mm vs. 23.84 ± 0.84 mm; *p* < 0.01) and thinner lens thickness (3.502 ± 0.187 mm vs. 4.017 ± 0.401 mm; *p* < 0.0001).

#### Image Quality Assessment

Image acquisition achieved high signal strength across age groups and, notably, no pediatric scans were excluded for quality reasons; younger children recorded slightly higher ONH signal strength than older children (9.261 ± 1.054 vs. 8.286 ± 0.914; *p* < 0.01), underscoring practical feasibility.

### 3.2. OCT Findings

#### 3.2.1. ONH Analysis (Table 2)

Structural OCT revealed no significant RNFL difference between children and adults for any measurements.

Within pediatric subgroups, younger children showed significantly thicker RNFL at clock hour 5 (111.30 ± 20.35 μm vs. 95.79 ± 24.00 μm, *p* < 0.05)) and hour 6 (142.95 ± 22.66 μm vs. 119.71 ± 28.73 μm, *p* < 0.01) compared to older children.

**Figure 1:**
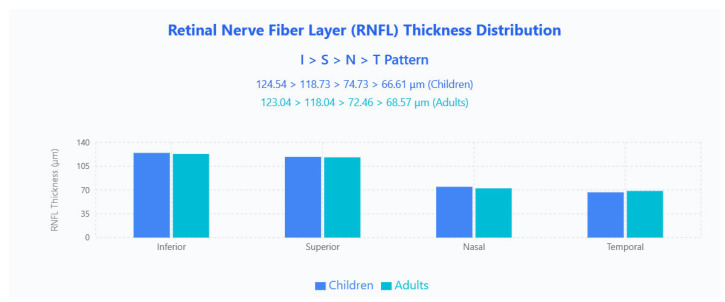
RNFL thickness distribution showing the classic ISNT pattern in both pediatric and adult populations. Bar chart displays mean RNFL thickness values across four quadrants (Inferior, Superior, Nasal, Temporal) for children (blue bars) and adults (cyan bars).

The RNFL thickness distribution followed the classic ISNT pattern (Inferior > Superior > Nasal > Temporal) in both age groups (Figure 1).

#### 3.2.2. Macula Analysis (Table 3)

##### MT

When comparing children to adults, macular analysis identified consistent age-related differences: total macular thickness was significantly lower in children (251.67 ± 21.32 μm) versus adults (270.36 ± 17.02 μm; *p* < 0.001), with all parafoveal quadrants showing similar significance (parafoveal superior *p* < 0.001, parafoveal nasal *p* < 0.001, parafoveal inferior *p* < 0.001, parafoveal temporal *p* < 0.001), whereas perifoveal measures remained comparable. Additionally, no significant differences in any macular parameters were observed within the pediatric subgroups or within the adult subgroups.

**Figure 2:**
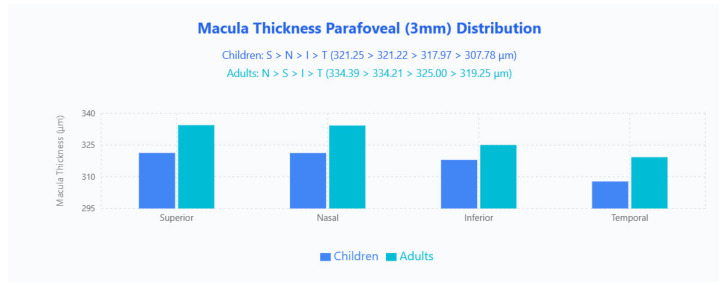
MT parafoveal (3 mm) distribution in pediatric and adult populations. Bar chart displays mean macular thickness values across four quadrants (Superior, Nasal, Inferior, Temporal) for children (blue bars) and adults (cyan bars).

Parafoveal 3 × 3 mm sectoral patterns contrasted by age (Figure 2): children demonstrated an S > N > I > T ordering (321.25 μm, 321.22 μm, 317.97 μm, 307.78 μm), whereas adults showed N > S > I > T (334.39 μm, 334.21 μm, 325.00 μm, 319.25 μm), with adults thicker in every quadrant.

**Figure 3:**
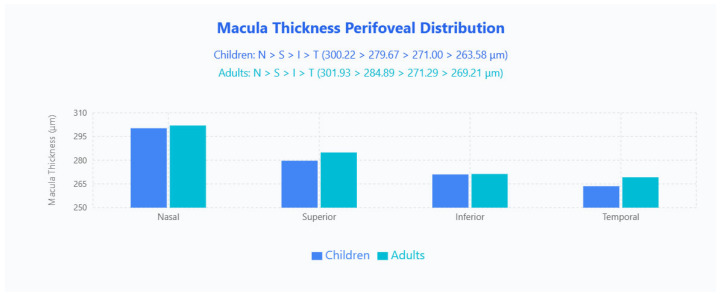
Macula thickness perifoveal distribution in pediatric and adult populations. Bar chart displays mean macular thickness values across four quadrants (Nasal, Superior, Inferior, Temporal) for children (blue bars) and adults (cyan bars).

Perifoveal 6 × 6 mm sectors followed an identical N > S > I > T pattern across both groups (children: 300.22 μm, 279.67 μm, 271.00 μm, 263.58 μm; adults: 301.93 μm, 284.89 μm, 271.29 μm, 269.21 μm), and here adults were generally thicker except the inferior quadrant, which was effectively unchanged (Figure 3).

##### GCIPL

**Figure 4:**
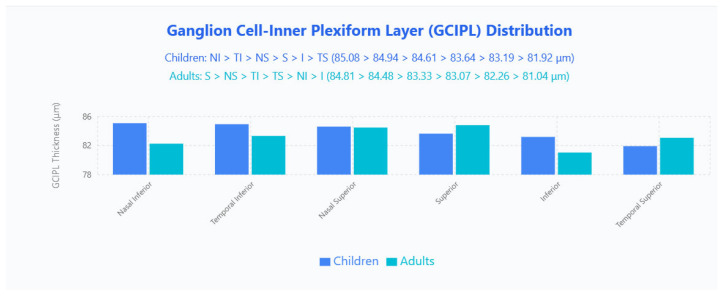
GCIPL distribution in pediatric and adult populations. Bar chart displays mean GCIPL thickness values across six sectors (Nasal Inferior, Temporal Inferior, Nasal Superior, Superior, Inferior, Temporal Superior) for children (blue bars) and adults (cyan bars).

No significant differences in mean GCIPL thickness were found between groups. Regionally, children displayed a nasal-inferior predominance (NI > TI > NS > S > I > TS; 85.08, 84.94, 84.61, 83.64, 83.19, 81.92 μm), whereas adults showed a superior predominance (S > NS > TI > TS > NI > I; 84.81, 84.48, 83.33, 83.07, 82.26, 81.04 μm) (Figure 4).

### 3.3. OCT-A Findings

#### 3.3.1. ONH (Table 4)

Children demonstrated significantly higher PF values compared to adults in multiple regions: complete outer region (45.74 ± 1.66% vs. 44.33 ± 1.26%, *p* < 0.001), nasal (43.94 ± 3.00% vs. 42.09 ± 1.61%, *p* < 0.01), inferior (46.69 ± 2.67% vs. 44.99 ± 1.57%, *p* < 0.01), and temporal (47.81 ± 1.95% vs. 46.54 ± 2.38%, *p* < 0.05). No significant differences were found for FI parameters. Within the pediatric cohort, younger children (1–9 years) showed higher PF than older children (10–17 years) in the complete outer region (46.29 ± 1.30% vs. 44.83 ± 1.82%, *p* < 0.05), superior (44.74 ± 1.96% vs. 43.26 ± 2.14%, *p* < 0.05) and inferior regions (47.80 ± 1.69% vs. 45.19 ± 3.06%, *p* < 0.01).

##### ONH PF Distribution

**Figure 5:**
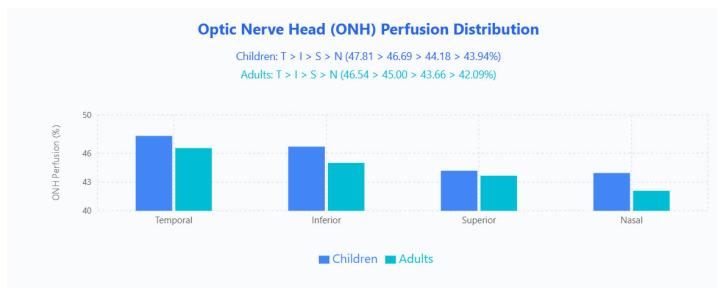
ONH PF distribution in pediatric and adult populations. Bar chart displays mean ONH perfusion values across four quadrants (Temporal, Inferior, Superior, Nasal) for children (blue bars) and adults (cyan bars).

Regional PF retained an identical hierarchy in both age groups (temporal > inferior > superior > nasal): children 47.81% (T), 46.69% (I), 44.18% (S), 43.94% (N); adults 46.54% (T), 45.00% (I), 43.66% (S), 42.09% (N), with children higher in absolute PF across all quadrants (Figure 5).

##### ONH FI Distribution

**Figure 6:**
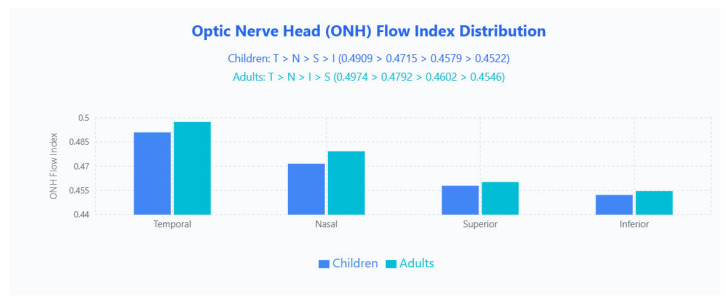
ONH FI distribution in pediatric and adult populations. Bar chart displays mean ONH FI values across four quadrants (Temporal, Nasal, Superior, Inferior) for children (blue bars) and adults (cyan bars).

FI patterns differed by age: children followed T > N > S > I (0.490, 0.472, 0.458, 0.452) while adults showed T > N > I > S (0.497, 0.479, 0.460, 0.455); the temporal quadrant remained highest in both groups, whereas the swapped ranking of inferior and superior quadrants indicates an age-related redistribution of perfusion intensity (Figure 6).

#### 3.3.2. Macula Analysis (Table 5)

No significant differences were observed between children and adults for any macular OCT-A parameters, including VD, PD, or FAZ measurements.

Within the pediatric cohort, however, younger children consistently exhibited higher VD and PD across both 3 × 3 mm and 6 × 6 mm scans; for example, in 3 × 3 mm measures younger children had higher parafoveal complete VD (22.91 ± 0.76% vs. 21.67 ± 1.46%, *p* < 0.01) and complete area VD (21.81 ± 0.85% vs. 20.65 ± 1.22%, *p* < 0.01).

**Table 1:** Study Collective Characteristics. Values presented as mean ± SD. Age subgroups: children (1–9 vs. 10–17 years), adults (19–31 vs. ≥32 years). *p*-Values represent intragroup (children/adults) and intergroup (children vs. adults) comparisons. Statistically significant differences (*p* < 0.05) marked with asterisk (*). Effect sizes (Cohen’s d) are provided for significant comparisons to quantify the magnitude of observed differences. Abbreviations: LogMAR, logarithm of minimum angle of resolution; RNFL, retinal nerve fiber layer; CDR, cup-to-disk ratio.

Description	Unit	Children (Overall)	1–9 Years	10–17 Years	*p*-Value/Effect Size (Children)	Adults (Overall)	19–31 Years	≥32 Years	*p*-Value/Effect Size (Adults)	*p*-Value/Effect Size (Overall)
Age	Years	10.20 ± 4.42 (n = 37)	7.25 ± 1.72 (n = 23)	15.05 ± 2.90 (n = 14)	*p* < 0.0001 * d = 3.270	37.78 ± 12.49 (n = 28)	28.87 ± 2.55 (n = 14)	46.70 ± 12.11 (n = 14)	*p* < 0.0001 * d = 2.038	*p* < 0.0001 * d = 2.944
Gender	Female = 1 Male = 2	1: 56.8% (n = 21) 2: 43.2% (n = 16)	1: 47.8 (n = 11) 2: 52.3% (n = 12)	1: 71.4% (n = 10) 2: 71.4% (n = 4)	*p* = 0.288	1: 82.1% (n = 23) 2: 17.9% (n = 5)	1: 71.4% (n = 10) 2: 28.6% (n = 4)	1: 92.9% (n = 13) 2: 7.1% (n = 1)	*p* = 0.326	*p* = 0.058
Eye	OD = 1 OS = 2	1: 36 (97.3%) 2: 1 (2.7%)	1: 22 (95.7%) 2: 1 (4.3%)	1: 14 (100%) 2: 0 (0.0%)	*p* = 1.000	1: 25 (89.3%) 2: 3 (10.7%)	1: 14 (100%) 2: 0 (0.0%)	1: 11 (78.6%) 2: 3 (21.4%)	*p* = 0.222	*p* = 0.307
Axial Length	mm	22.90 ± 0.99 (n = 36)	22.49 ± 0.757 (n = 22)	23.55 ± 0.981 (n = 14)	*p* < 0.001 * d = 1.213	23.84 ± 0.84 (n = 28)	23.826 ± 0.698 (n = 14)	23.853 ± 0.985 (n = 14)	*p* = 0.934 d = 0.032	*p* < 0.001 * d = 1.024
Spherical Equivalent Refraction	dpt	−0.146 ± 1.813 (n = 36)	0.0682 ± 0.3198 (n = 22)	−0.482 ± 1.668 (n = 14)	*p* = 0.166 d = 0.458	−1.125 ± 2.144 (n = 28)	−0.2500 ± 0.6124 (n = 14)	−2.000 ± 2.742 (n = 14)	*p* = 0.132 d = 0.881	*p* < 0.05 * d = 0.577
Anterior Chamber Depth	mm	4.096 ± 2.977 (n = 36)	3.5509 ± 0.2374 (n = 22)	4.95 ± 4.74 (n = 14)	*p* = 0.080 d = 0.417	4.19 ± 3.88 (n = 28)	4.96 ± 5.46 (n = 14)	3.426 ± 0.444 (n = 14)	*p* = 0.730 d = 0.396	*p* = 0.256 d = 0.028
Lens Thickness	mm	3.502 ± 0.187 (n = 32)	3.5200 ± 0.1190 (n = 19)	3.4762 ± 0.2601 (n = 13)	*p* = 0.578 d = 0.217	4.017 ± 0.401 (n = 28)	3.798 ± 0.212 (n = 14)	4.24 ± 0.43 (n = 14)	*p* < 0.01 * d = 1.281	*p* < 0.0001 * d = 1.644
Central Corneal Thickness	μm	551.0 ± 34.5 (n = 32)	552.4 ± 36.2 (n = 19)	548.8 ± 33.2 (n = 13)	*p* = 0.779 d = 0.103	552.50 ± 28.65 (n = 28)	540.43 ± 30.16 (n = 14)	564.57 ± 21.95 (n = 14)	*p* < 0.05 * d = 0.915	*p* = 0.854 d = 0.048
White-to-White	mm	15.38 ± 18.18 (n = 32)	17.58 ± 23.59 (n = 19)	12.169 ± 0.444 (n = 13)	*p* = 0.878 d = 0.324	12.257 ± 0.398 (n = 28)	12.450 ± 0.363 (n = 14)	12.06 ± 0.341 (n = 14)	*p* < 0.01 * d = 1.095	*p* = 0.517 d = 0.243
Pupil Diameter	mm	5.700 ± 1.136 (n = 32)	5.32 ± 1.02 (n = 19)	6.25 ± 1.10 (n = 13)	*p* < 0.05 * d = 0.878	5.164 ± 1.307 (n = 28)	5.564 ± 0.868 (n = 14)	4.764 ± 1.566 (n = 14)	*p* = 0.107 d = 0.632	*p* = 0.095 d = 0.437
CDR Clinical		0.1946 ± 0.1929 (n = 37)	0.2000 ± 0.2153 (n = 23)	0.1857 ± 0.1556 (n = 14)	*p* = 0.859 d = 0.076	0.1357 ± 0.0826 (n = 28)	0.1500 ± 0.0941 (n = 14)	0.1214 ± 0.0699 (n = 14)	*p* = 0.453 d = 0.345	*p* = 0.277 d = 0.397
Visual Acuity logMAR	LogMAR	0.01714 ± 0.04528 (n = 35)	0.02727 ± 0.04558 (n = 22)	0.0000 ± 0.0408 (n = 13)	*p* = 0.097 d = 0.630	0.00357 ± 0.01890 (n = 28)	0.00714 ± 0.02673 (n = 14)	0 ± 0 (n = 14)	*p* = 0.353 d = 0.378	*p* = 0.130 d = 0.391
Intraocular Pressure	mmHg	16.86 ± 3.79 (n = 37)	17.35 ± 3.59 (n = 23)	16.07 ± 4.12 (n = 14)	*p* = 0.328 d = 0.330	15.64 ± 3.95 (n = 28)	16.43 ± 4.15 (n = 14)	14.86 ± 3.72 (n = 14)	*p* = 0.301 d = 0.399	*p* = 0.211 d = 0.316

**Table 2:** OCT measurements of retinal nerve fiber layer (RNFL) thickness. Values presented as mean ± SD. Age subgroups: children (1–9 vs. 10–17 years), adults (19–31 vs. ≥32 years). *p*-Values represent intragroup (children/adults) and intergroup (children vs. adults) comparisons. Statistically significant differences (*p* < 0.05) marked with asterisk (*). Effect sizes (Cohen’s d) are provided for significant comparisons to quantify the magnitude of observed differences. Abbreviations: RNFL, retinal nerve fiber layer.

Description	Unit	Children (Overall)	1–9 Years	10–17 Years	*p*-Value/Effect Size (Children)	Adults (Overall)	19–31 Years	≥32 Years	*p*-Value/Effect Size (Adults)	*p*-Value/Effect Size (Overall)
Optic Disc Cube Signal Strength		8.892 ± 1.100 (n = 37)	9.261 ± 1.054 (n = 23)	8.286 ± 0.914 (n = 14)	*p* < 0.01 * d = 0.989	8.679 ± 0.772 (n = 28)	8.857 ± 0.535 (n = 14)	8.509 ± 0.941 (n = 14)	*p* = 0.244 d = 0.467	*p* = 0.226 d = 0.224
Average RNFL Thickness	μm	96.11 ± 11.35 (n = 37)	97.39 ± 9.94 (n = 23)	94.00 ± 13.49 (n = 14)	*p* = 0.386 d = 0.286	95.57 ± 8.69 (n = 28)	95.14 ± 8.62 (n = 14)	96.00 ± 9.06 (n = 14)	*p* = 0.800 d = 0.097	*p* = 0.836 d = 0.053
Vertical C/D Ratio		0.4116 ± 0.1694 (n = 37)	0.4300 ± 0.1906 (n = 23)	0.3814 ± 0.1281 (n = 14)	*p* = 0.405 d = 0.299	0.3668 ± 0.1910 (n = 28)	0.4343 ± 0.1936 (n = 14)	0.2993 ± 0.1688 (n = 14)	*p* = 0.060 d = 0.743	*p* = 0.321 d = 0.248
RNFL Superior Quadrant	μm	118.73 ± 18.64 (n = 37)	118.74 ± 16.58 (n = 23)	118.71 ± 22.28 (n = 14)	*p* = 0.997 d = 0.001	118.04 ± 16.11 (n = 28)	118.57 ± 17.21 (n = 14)	117.50 ± 15.57 (n = 14)	*p* = 0.864 d = 0.065	*p* = 0.875 d = 0.040
RNFL Nasal Quadrant	μm	74.73 ± 14.43 (n = 37)	77.22 ± 14.40 (n = 23)	70.64 ± 14.03 (n = 14)	*p* = 0.183 d = 0.463	72.46 ± 6.32 (n = 28)	71.00 ± 4.69 (n = 14)	73.93 ± 7.50 (n = 14)	*p* = 0.226 d = 0.468	*p* = 0.398 d = 0.203
RNFL Inferior Quadrant	μm	124.54 ± 16.81 (n = 37)	128.17 ± 13.15 (n = 23)	118.57 ± 20.68 (n = 14)	*p* = 0.092 d = 0.554	123.04 ± 15.69 (n = 28)	123.64 ± 13.79 (n = 14)	122.43 ± 17.90 (n = 14)	*p* = 0.842 d = 0.076	*p* = 0.714 d = 0.093
RNFL Temporal Quadrant	μm	66.61 ± 9.00 (n = 37)	65.48 ± 9.83 (n = 23)	68.21 ± 7.46 (n = 14)	*p* = 0.377 d = 0.313	68.57 ± 14.48 (n = 28)	66.79 ± 10.69 (n = 14)	70.36 ± 17.74 (n = 14)	*p* = 0.730 d = 0.244	*p* = 0.858 d = 0.171
RNFL Hour 12	μm	121.27 ± 31.38 (n = 37)	123.35 ± 30.86 (n = 23)	117.9 ± 33.1 (n = 14)	*p* = 0.613 d = 0.172	114.36 ± 28.05 (n = 28)	113.57 ± 22.80 (n = 14)	115.1 ± 33.4 (n = 14)	*p* = 0.945 d = 0.055	*p* = 0.382 d = 0.232
RNFL Hour 1	μm	107.03 ± 19.52 (n = 37)	105.57 ± 17.04 (n = 23)	109.43 ± 23.54 (n = 14)	*p* = 0.567 d = 0.188	108.25 ± 22.93 (n = 28)	108.14 ± 26.24 (n = 14)	108.36 ± 20.08 (n = 14)	*p* = 0.981 d = 0.009	*p* = 0.817 d = 0.057
RNFL Hour 2	μm	98.24 ± 21.65 (n = 37)	103.57 ± 20.46 (n = 23)	89.50 ± 21.37 (n = 14)	*p* = 0.054 d = 0.672	91.36 ± 10.55 (n = 28)	91.86 ± 10.68 (n = 14)	90.86 ± 10.78 (n = 14)	*p* = 0.807 d = 0.093	*p* = 0.097 d = 0.404
RNFL Hour 3	μm	57.35 ± 11.08 (n = 37)	59.09 ± 12.66 (n = 23)	54.50 ± 7.40 (n = 14)	*p* = 0.347 d = 0.443	57.32 ± 9.18 (n = 28)	55.36 ± 8.18 (n = 14)	59.29 ± 10.00 (n = 14)	*p* = 0.265 d = 0.430	*p* = 0.931 d = 0.003
RNFL Hour 4	μm	69.30 ± 16.48 (n = 37)	69.70 ± 15.20 (n = 23)	68.64 ± 18.99 (n = 14)	*p* = 0.854 d = 0.061	67.82 ± 11.68 (n = 28)	65.57 ± 8.72 (n = 14)	70.07 ± 14.01 (n = 14)	*p* = 0.317 d = 0.386	*p* = 0.688 d = 0.103
RNFL Hour 5	μm	105.43 ± 22.79 (n = 37)	111.30 ± 20.35 (n = 23)	95.79 ± 24.00 (n = 14)	*p* < 0.05 * d = 0.697	101.82 ± 25.84 (n = 28)	95.71 ± 16.33 (n = 14)	107.9 ± 32.3 (n = 14)	*p* = 0.250 d = 0.478	*p* = 0.546 d = 0.148
RNFL Hour 6	μm	134.16 ± 27.24 (n = 37)	142.95 ± 22.66 (n = 23)	119.71 ± 28.73 (n = 14)	*p* < 0.01 * d = 0.898	134.89 ± 25.81 (n = 28)	136.07 ± 21.63 (n = 14)	133.71 ± 30.22 (n = 14)	*p* = 0.814 d = 0.090	*p* = 0.913 d = 0.028
RNFL Hour 7	μm	133.92 ± 19.83 (n = 37)	130.22 ± 20.18 (n = 23)	140.00 ± 18.33 (n = 14)	*p* = 0.148 d = 0.507	132.82 ± 24.04 (n = 28)	139.71 ± 20.74 (n = 14)	125.93 ± 25.85 (n = 14)	*p* = 0.132 d = 0.588	*p* = 0.841 d = 0.050
RNFL Hour 8	μm	71.46 ± 13.98 (n = 37)	69.35 ± 14.41 (n = 23)	74.93 ± 13.00 (n = 14)	*p* = 0.244 d = 0.407	71.89 ± 17.93 (n = 28)	70.50 ± 12.11 (n = 14)	73.29 ± 22.56 (n = 14)	*p* = 0.927 d = 0.154	*p* = 0.963 d = 0.027
RNFL Hour 9	μm	50.92 ± 7.05 (n = 37)	50.91 ± 7.46 (n = 23)	50.93 ± 6.58 (n = 14)	*p* = 0.995 d = 0.002	52.86 ± 8.81 (n = 28)	51.64 ± 7.38 (n = 14)	54.07 ± 10.19 (n = 14)	*p* = 0.504 d = 0.273	*p* = 0.691 d = 0.243
RNFL Hour 10	μm	76.95 ± 13.46 (n = 37)	75.74 ± 14.32 (n = 23)	78.93 ± 12.15 (n = 14)	*p* = 0.397 d = 0.240	82.11 ± 19.77 (n = 28)	78.29 ± 17.06 (n = 14)	85.93 ± 22.12 (n = 14)	*p* = 0.222 d = 0.387	*p* = 0.628 d = 0.305
RNFL Hour 11	μm	128.00 ± 17.53 (n = 37)	127.26 ± 14.67 (n = 23)	129.21 ± 22.01 (n = 14)	*p* = 0.747 d = 0.104	130.93 ± 18.58 (n = 28)	133.50 ± 17.77 (n = 14)	128.36 ± 19.68 (n = 14)	*p* = 0.475 d = 0.274	*p* = 0.518 d = 0.162

**Table 3:** OCT measurements of macular thickness (MT) and ganglion cell layer analysis (GCIPL) in different regions of the macula. Values presented as mean ± SD. Age subgroups: children (1–9 vs. 10–17 years), adults (19–31 vs. ≥32 years). *p*-Values represent intragroup (children/adults) and intergroup (children vs. adults) comparisons. Statistically significant differences (*p* < 0.05) marked with asterisk (*). Effect sizes (Cohen’s d) are provided for significant comparisons to quantify the magnitude of observed differences. Abbreviations: MT, macular thickness; GCIPL, ganglion cell-inner plexiform layer.

Description	Unit	Children (Overall)	1–9 Years	10–17 Years	*p*-Value/Effect Size (Children)	Adults (Overall)	19–31 Years	≥32 Years	*p*-Value/Effect Size (Adults)	*p*-Value/Effect Size (Overall)
Macular Cube Signal Strength	Number	9.333 ± 0.894 (n = 36)	9.500 ± 0.802 (n = 22)	9.071 ± 0.997 (n = 14)	*p* = 0.164 d = 0.474	9.643 ± 0.678 (n = 28)	9.571 ± 0.756 (n = 14)	9.714 ± 0.611 (n = 14)	*p* = 0.649 d = 0.208	*p* = 0.121 d = 0.390
Macular Cube Total Thickness	μm	251.67 ± 21.32 (n = 36)	246.27 ± 16.91 (n = 22)	260.14 ± 25.21 (n = 14)	*p* = 0.056 d = 0.646	270.36 ± 17.02 (n = 28)	266.29 ± 14.75 (n = 14)	274.43 ± 18.67 (n = 14)	*p* = 0.212 d = 0.484	*p* < 0.001 * d = 0.969
MT Parafoveal Superior	μm	321.25 ± 10.57 (n = 36)	319.59 ± 10.34 (n = 22)	323.86 ± 10.78 (n = 14)	*p* = 0.243 d = 0.404	334.21 ± 13.54 (n = 28)	332.29 ± 17.46 (n = 14)	336.14 ± 8.24 (n = 14)	*p* = 0.464 d = 0.283	*p* < 0.0001 * d = 1.068
MT Parafoveal Nasal	μm	321.22 ± 11.92 (n = 36)	319.00 ± 11.98 (n = 22)	324.71 ± 11.37 (n = 14)	*p* = 0.164 d = 0.489	334.39 ± 14.07 (n = 28)	331.71 ± 16.81 (n = 14)	337.07 ± 10.64 (n = 14)	*p* = 0.323 d = 0.381	*p* < 0.001 * d = 1.010
MT Parafoveal Inferior	μm	317.97 ± 10.46 (n = 36)	315.82 ± 10.63 (n = 22)	321.36 ± 9.59 (n = 14)	*p* = 0.123 d = 0.547	325.00 ± 16.28 (n = 28)	322.86 ± 22.10 (n = 14)	327.14 ± 7.22 (n = 14)	*p* = 1.000 d = 0.261	*p* < 0.01 * d = 0.514
MT Parafoveal Temporal	μm	307.78 ± 10.50 (n = 36)	306.00 ± 10.40 (n = 22)	310.57 ± 10.40 (n = 14)	*p* = 0.207 d = 0.440	319.25 ± 17.50 (n = 28)	315.14 ± 22.60 (n = 14)	323.36 ± 9.43 (n = 14)	*p* = 0.476 d = 0.474	*p* < 0.001 * d = 0.795
MT Perifoveal Superior	μm	279.67 ± 12.64 (n = 36)	279.77 ± 10.53 (n = 22)	279.50 ± 15.84 (n = 14)	*p* = 0.951 d = 0.020	284.89 ± 13.40 (n = 28)	283.00 ± 16.66 (n = 14)	286.79 ± 9.35 (n = 14)	*p* = 0.465 d = 0.280	*p* = 0.115 d = 0.401
MT Perifoveal Nasal	μm	300.22 ± 14.05 (n = 36)	299.18 ± 12.22 (n = 22)	301.86 ± 16.90 (n = 14)	*p* = 0.585 d = 0.181	301.93 ± 14.75 (n = 28)	300.64 ± 18.51 (n = 14)	303.21 ± 9.27 (n = 14)	*p* = 0.653 d = 0.172	*p* = 0.639 d = 0.118
MT Perifoveal Inferior	μm	271.00 ± 10.70 (n = 36)	270.73 ± 9.17 (n = 22)	271.43 ± 13.12 (n = 14)	*p* = 0.782 d = 0.062	271.29 ± 12.87 (n = 28)	271.36 ± 17.11 (n = 14)	271.21 ± 7.16 (n = 14)	*p* = 0.977 d = 0.011	*p* = 0.709 d = 0.024
MT Perifoveal Temporal	μm	263.58 ± 10.41 (n = 36)	263.73 ± 8.92 (n = 22)	263.36 ± 12.76 (n = 14)	*p* = 0.919 d = 0.034	269.21 ± 17.29 (n = 28)	265.86 ± 16.89 (n = 14)	272.57 ± 17.64 (n = 14)	*p* = 0.476 d = 0.389	*p* = 0.093 d = 0.395
GCIPL Total	μm	83.83 ± 5.80 (n = 36)	83.50 ± 4.62 (n = 22)	84.36 ± 7.47 (n = 14)	*p* = 0.672 d = 0.138	83.15 ± 5.43 (n = 27)	84.43 ± 6.09 (n = 14)	81.77 ± 4.44 (n = 13)	*p* = 0.209 d = 0.499	*p* = 0.635 d = 0.122
GCIPL Superior	μm	83.64 ± 6.15 (n = 36)	83.05 ± 4.71 (n = 22)	84.57 ± 8.04 (n = 14)	*p* = 0.528 d = 0.232	84.81 ± 6.38 (n = 27)	85.93 ± 6.90 (n = 14)	83.62 ± 5.80 (n = 13)	*p* = 0.357 d = 0.363	*p* = 0.463 d = 0.188
GCIPL Nasal Superior	μm	84.61 ± 7.17 (n = 36)	85.00 ± 5.10 (n = 22)	84.00 ± 9.78 (n = 14)	*p* = 0.728 d = 0.128	84.48 ± 6.14 (n = 27)	85.93 ± 6.60 (n = 14)	82.92 ± 5.41 (n = 13)	*p* = 0.210 d = 0.498	*p* = 0.940 d = 0.019
GCIPL Nasal Inferior	μm	85.08 ± 6.38 (n = 36)	84.73 ± 5.70 (n = 22)	85.64 ± 7.52 (n = 14)	*p* = 0.681 d = 0.137	82.26 ± 6.22 (n = 27)	84.14 ± 6.92 (n = 14)	80.23 ± 4.83 (n = 13)	*p* = 0.103 d = 0.656	*p* = 0.084 d = 0.448
GCIPL Inferior	μm	83.19 ± 6.55 (n = 36)	83.27 ± 4.69 (n = 22)	83.07 ± 8.94 (n = 14)	*p* = 0.807 d = 0.028	81.04 ± 6.19 (n = 27)	82.00 ± 7.06 (n = 14)	80.00 ± 5.18 (n = 13)	*p* = 0.412 d = 0.323	*p* = 0.095 d = 0.338
GCIPL Temporal Inferior	μm	84.94 ± 5.82 (n = 36)	84.18 ± 4.52 (n = 22)	86.14 ± 7.46 (n = 14)	*p* = 0.332 d = 0.318	83.33 ± 5.46 (n = 27)	84.64 ± 6.54 (n = 14)	81.92 ± 3.77 (n = 13)	*p* = 0.202 d = 0.510	*p* = 0.269 d = 0.285
GCIPL Temporal Superior	μm	81.92 ± 5.51 (n = 36)	81.23 ± 4.21 (n = 22)	83.00 ± 7.15 (n = 14)	*p* = 0.354 d = 0.302	83.07 ± 5.12 (n = 27)	83.64 ± 5.93 (n = 14)	82.46 ± 4.24 (n = 13)	*p* = 0.330 d = 0.229	*p* = 0.161 d = 0.218

**Table 4:** OCT-A measurements of perfusion (PF) and flow index (FI) in different regions of the ONH. Values presented as mean ± SD. Age subgroups: children (1–9 vs. 10–17 years), adults (19–31 vs. ≥32 years). *p*-Values represent intragroup (children/adults) and intergroup (children vs. adults) comparisons. Statistically significant differences (*p* < 0.05) marked with asterisk (*). Effect sizes (Cohen’s d) are provided for significant comparisons to quantify the magnitude of observed differences. Abbreviations: OCT-A, optical coherence tomography angiography; PF, perfusion; FI, flow index; ONH, optic nerve head.

Description	Unit	Children (Overall)	1–9 Years	10–17 Years	*p*-Value/Effect Size (Children)	Adults (Overall)	19–31 Years	≥32 Years	*p*-Value/Effect Size (Adults)	*p*-Value/Effect Size (Overall)
ONH Signal Strength	Number	9.87 ± 0.41 (n = 37)	9.87 ± 0.46 (n = 23)	9.86 ± 0.36 (n = 14)	*p* = 0.663 d = 0.030	9.59 ± 0.74 (n = 27)	9.71 ± 0.61 (n = 14)	9.46 ± 0.88 (n = 13)	*p* = 0.507 d = 0.334	*p* = 0.100 d = 0.449
ONH PF—Complete Outer Region	%	45.74 ± 1.66 (n = 37)	46.29 ± 1.30 (n = 23)	44.83 ± 1.82 (n = 14)	*p* < 0.05 * d = 0.920	44.33 ± 1.26 (n = 27)	44.51 ± 1.23 (n = 14)	44.13 ± 1.32 (n = 13)	*p* = 0.452 d = 0.294	*p* < 0.001 * d = 0.949
ONH PF—Superior	%	44.18 ± 2.13 (n = 37)	44.74 ± 1.96 (n = 23)	43.26 ± 2.14 (n = 14)	*p* < 0.05 * d = 0.716	43.66 ± 2.13 (n = 27)	43.91 ± 2.04 (n = 14)	43.39 ± 2.28 (n = 13)	*p* = 0.593 d = 0.245	*p* = 0.190 d = 0.244
ONH PF—Nasal	%	43.94 ± 3.00 (n = 35)	44.65 ± 3.09 (n = 22)	42.75 ± 2.51 (n = 13)	*p* = 0.069 d = 0.676	42.09 ± 1.61 (n = 27)	42.24 ± 1.65 (n = 14)	41.92 ± 1.61 (n = 13)	*p* = 0.615 d = 0.196	*p* < 0.01 * d = 0.770
ONH PF—Inferior	%	46.69 ± 2.67 (n = 33)	47.80 ± 1.69 (n = 19)	45.19 ± 3.06 (n = 14)	*p* < 0.01 * d = 1.056	45.00 ± 1.57 (n = 27)	45.14 ± 1.64 (n = 14)	44.84 ± 1.54 (n = 13)	*p* = 0.625 d = 0.191	*p* < 0.01 * d = 0.773
ONH PF—Temporal	%	47.81 ± 1.95 (n = 36)	47.70 ± 1.87 (n = 22)	47.96 ± 2.14 (n = 14)	*p* = 0.708 d = 0.127	46.54 ± 2.38 (n = 27)	46.67 ± 2.15 (n = 14)	46.40 ± 2.70 (n = 13)	*p* = 0.774 d = 0.111	*p* < 0.05 * d = 0.581
ONH FI—Complete Outer Region	**-**	0.467 ± 0.026 (n = 37)	0.468 ± 0.025 (n = 23)	0.467 ± 0.027 (n = 14)	*p* = 0.947 d = 0.022	0.622 ± 0.772 (n = 27)	0.474 ± 0.018 (n = 14)	0.782 ± 1.112 (n = 13)	*p* = 0.903 d = 0.391	*p* = 0.256 d = 0.283
ONH FI—Superior	**-**	0.458 ± 0.040 (n = 37)	0.454 ± 0.027 (n = 23)	0.465 ± 0.056 (n = 14)	*p* = 0.913 d = 0.241	0.455 ± 0.019 (n = 27)	0.456± 0.016 (n = 14)	0.452 ± 0.022 (n = 13)	*p* = 1.000 d = 0.245	*p* = 0.812 d = 0.105
ONH FI—Nasal	**-**	0.472± 0.026 (n = 35)	0.473± 0.025 (n = 22)	0.468± 0.028 (n = 13)	*p* = 0.626 d = 0.169	0.479 ± 0.023 (n = 27)	0.4788± 0.022 (n = 14)	0.479 ± 0.025 (n = 13)	*p* = 0.928 d = 0.035	*p* = 0.235 d = 0.310
ONH FI—Inferior	**-**	0.452 ± 0.023 (n = 33)	0.452 ± 0.024 (n = 19)	0.451 ± 0.02 (n = 14)	*p* = 0.959 d = 0.018	0.460 ± 0.015 (n = 27)	0.459 ± 0.015 (n = 14)	0.461 ± 0.014 (n = 13)	*p* = 0.719 d = 0.141	*p* = 0.133 d = 0.404
ONH FI—Temporal	**-**	0.490 ± 0.03 (n = 36)	0.491± 0.03 (n = 22)	0.490 ± 0.041 (n = 14)	*p* = 0.954 d = 0.019	0.497 ± 0.025 (n = 27)	0.496 ± 0.023 (n = 14)	0.498 ± 0.02 (n = 13)	*p* = 0.840 d = 0.078	*p* = 0.407 d = 0.217

**Table 5:** OCT-A measurements of vessel density (VD), perfusion density (PD), and foveal avascular zone (FAZ) parameters in macular regions. Values presented as mean ± SD. Age subgroups: children (1–9 vs. 10–17 years), adults (19–31 vs. ≥32 years). *p*-Values represent intragroup (children/adults) and intergroup (children vs. adults) comparisons. Statistically significant differences (*p* < 0.05) marked with asterisk (*). Effect sizes (Cohen’s d) are provided for significant comparisons to quantify the magnitude of observed differences. Abbreviations: OCT-A, optical coherence tomography angiography; VD, vessel density; PD, perfusion density; FAZ, foveal avascular zone.

Description	Unit	Children (Overall)	1–9 Years	10–17 Years	*p*-Value/Effect Size (Children)	Adults (Overall)	19–31 Years	≥32 Years	*p*-Value/Effect Size (Adults)	*p*-Value/Effect Size (Overall)
3 × 3 Signal Strength	Number	9.743 ± 0.611 (n = 35)	9.714 ± 0.644 (n = 21)	9.786 ± 0.579 (n = 14)	*p* = 0.739 d = 0.117	9.741 ± 0.594 (n = 27)	9.923 ± 0.277 (n = 13)	9.571 ± 0.756 (n = 14)	*p* = 0.162 d = 0.618	*p* = 0.924 d = 0.004
3 × 3 VD foveal	-	12.951 ± 3.118 (n = 35)	13.27 ± 3.29 (n = 21)	12.471 ± 2.887 (n = 14)	*p* = 0.465 d = 0.258	13.132 ± 2.885 (n = 28)	13.321 ± 2.969 (n = 14)	12.943 ± 2.896 (n = 14)	*p* = 0.736 d = 0.129	*p* = 0.814 d = 0.060
3 × 3 VD Parafoveal Complete	-	22.411 ± 1.234 (n = 35)	22.91 ± 0.76 (n = 21)	21.671 ± 1.456 (n = 14)	*p* < 0.01 * d = 1.063	22.257 ± 1.640 (n = 28)	22.086 ± 1.865 (n = 14)	22.429 ± 1.430 (n = 14)	*p* = 0.590 d = 0.206	*p* = 0.950 d = 0.106
3 × 3 VD Complete	-	21.349 ± 1.153 (n = 35)	21.81 ± 0.85 (n = 21)	20.65 ± 1.22 (n = 14)	*p* < 0.01 * d = 1.106	21.229 ± 1.630 (n = 28)	21.107 ± 1.847 (n = 14)	21.350 ± 1.439 (n = 14)	*p* = 0.701 d = 0.147	*p* = 0.734 d = 0.085
3 × 3 VD Parafoveal Superior	-	22.257 ± 1.589 (n = 35)	22.85 ± 1.07 (n = 21)	21.37 ± 1.85 (n = 14)	*p* < 0.05 * d = 0.976	21.964 ± 1.831 (n = 28)	21.614 ± 2.170 (n = 14)	22.314 ± 1.410 (n = 14)	*p* = 0.321 d = 0.383	*p* = 0.451 d = 0.171
3 × 3 VD Parafoveal Nasal	-	22.783 ± 0.993 (n = 35)	23.21 ± 0.79 (n = 21)	22.15 ± 0.94 (n = 14)	*p* < 0.01 * d = 1.210	22.543 ± 1.873 (n = 28)	22.500 ± 2.000 (n = 14)	22.586 ± 1.811 (n = 14)	*p* = 0.945 d = 0.045	*p* = 0.983 d = 0.160
3 × 3 VD Parafoveal Inferior	-	22.15 ± 1.63 (n = 35)	22.53 ± 1.33 (n = 21)	21.59 ± 1.91 (n = 14)	*p* = 0.138 d = 0.568	22.06 ± 1.82 (n = 28)	22.09 ± 1.97 (n = 14)	22.04± 1.74 (n = 14)	*p* = 0.936 d = 0.031	*p* = 0.906 d = 0.052
3 × 3 VD Parafoveal Temporal	-	22.48 ± 1.35 (n = 35)	23.06 ± 0.76 (n = 21)	21.62± 1.59 (n = 14)	*p* < 0.01 * d = 1.150	22.45 ± 1.59 (n = 28)	22.12 ± 1.65 (n = 14)	22.79 ± 1.53 (n = 14)	*p* = 0.279 d = 0.418	*p* = 0.761 d = 0.020
3 × 3 PD Central	%	22.90 ± 5.68 (n = 35)	23.72 ± 6.13 (n = 21)	21.66 ± 4.88 (n = 14)	*p* = 0.337 d = 0.371	24.29 ± 6.98 (n = 28)	24.35 ± 7.21 (n = 14)	24.22 ± 7.00 (n = 14)	*p* = 0.963 d = 0.018	*p* = 0.410 d = 0.218
3 × 3 PD Parafoveal Complete	%	40.26 ± 2.18 (n = 35)	41.01 ± 1.45 (n = 21)	39.14 ± 2.65 (n = 14)	*p* < 0.01 * d = 0.876	39.56 ± 2.72 (n = 28)	39.24 ± 3.06 (n = 14)	39.88 ± 2.41 (n = 14)	*p* = 0.747 d = 0.231	*p* = 0.395 d = 0.282
3 × 3 PD Complete	%	38.28 ± 2.07 (n = 35)	39.01 ± 1.68 (n = 21)	37.18 ± 2.17 (n = 14)	*p* < 0.01 * d = 0.948	37.78 ± 2.77 (n = 28)	37.42 ± 3.01 (n = 14)	38.14 ± 2.58 (n = 14)	*p* = 0.765 d = 0.258	*p* = 0.740 d = 0.204
3 × 3 PD Parafoveal Superior	%	40.39 ± 2.57 (n = 35)	41.29 ± 2.32 (n = 21)	39.07 ± 2.41 (n = 14)	*p* < 0.05 * d = 0.932	39.60 ± 3.15 (n = 28)	38.76 ± 3.66 (n = 14)	40.44 ± 2.39 (n = 14)	*p* = 0.301 d = 0.545	*p* = 0.414 d = 0.276
3 × 3 PD Parafoveal Nasal	%	40.55 ± 1.68 (n = 35)	41.23± 1.19 (n = 21)	39.54 ± 1.83 (n = 14)	*p* < 0.01 * d = 1.094	39.536 ± 2.91 (n = 28)	39.35 ± 3.05 (n = 14)	39.72 ± 2.86 (n = 14)	*p* = 0.890 d = 0.126	*p* = 0.463 d = 0.427
3 × 3 PD Parafoveal Inferior	%	39.460 ± 3.06 (n = 35)	39.89 ± 2.54 (n = 21)	38.81 ± 3.71 (n = 14)	*p* = 0.490 d = 0.342	39.00 ± 3.08 (n = 28)	39.07 ± 3.12 (n = 14)	38.94 ± 3.16 (n = 14)	*p* = 0.818 d = 0.043	*p* = 0.557 d = 0.149
3 × 3 PD Parafoveal Temporal	%	40.51 ± 2.45 (n = 35)	41.41 ± 1.29 (n = 21)	39.16 ± 3.14 (n = 14)	*p* < 0.01 * d = 0.938	40.15 ± 2.49 (n = 28)	39.74 ± 2.71 (n = 14)	40.56 ± 2.28 (n = 14)	*p* = 0.394 d = 0.328	*p* = 0.472 d = 0.144
6 × 6 Signal Strength	Number	9.69 ± 0.63 (n = 36)	9.82 ± 0.50 (n = 22)	9.51 ± 0.77 (n = 14)	*p* = 0.295 d = 0.481	9.75 ± 0.52 (n = 28)	9.57 ± 0.67 (n = 14)	9.93± 0.27 (n = 14)	*p* = 0.072 d = 0.722	*p* = 0.978 d = 0.092
6 × 6 VD Central	-	11.07 ± 3.19 (n = 36)	11.13± 2.708 (n = 22)	10.98 ± 3.95 (n = 14)	*p* = 0.891 d = 0.045	11.61 ± 2.61 (n = 28)	11.67 ± 2.82 (n = 14)	11.55 ± 2.48 (n = 14)	*p* = 0.905 d = 0.046	*p* = 0.472 d = 0.185
6 × 6 VD Parafoveal Complete	-	18.40 ± 1.17 (n = 36)	18.62 ± 1.15 (n = 22)	18.06 ± 1.15 (n = 14)	*p* < 0.05 * d = 0.492	18.45 ± 0.94 (n = 28)	18.28 ± 1.13 (n = 14)	18.62 ± 0.70 (n = 14)	*p* = 0.344 d = 0.364	*p* = 0.812 d = 0.045
6 × 6 VD Perifoveal Complete	-	18.50 ± 1.07 (n = 36)	18.83± 0.69 (n = 22)	17.99 ± 1.37 (n = 14)	*p* < 0.05 * d = 0.771	18.65 ± 0.96 (n = 28)	18.53 ± 1.09 (n = 14)	18.77 ± 0.83 (n = 14)	*p* = 0.748 d = 0.251	*p* = 0.502 d = 0.145
6 × 6 VD Complete	-	18.25 ± 1.09 (n = 35)	18.56± 0.82 (n = 21)	17.79 ± 1.31 (n = 14)	*p* < 0.05 * d = 0.698	18.41 ± 0.95 (n = 28)	18.27 ± 1.11 (n = 14)	18.56 ± 0.79 (n = 14)	*p* = 0.439 d = 0.297	*p* = 0.575 d = 0.159
6 × 6 VD Parafoveal superior	-	18.40 ± 1.07 (n = 36)	18.65 ± 1.027 (n = 22)	18.02 ± 1.06 (n = 14)	*p* = 0.059 d = 0.597	18.38 ± 0.99 (n = 28)	18.16 ± 1.25 (n = 14)	18.61 ± 0.63 (n = 14)	*p* = 0.461 d = 0.455	*p* = 0.834 d = 0.020
6 × 6 VD Parafoveal nasal	-	18.36 ± 1.33 (n = 36)	18.48 ± 1.36 (n = 22)	18.16 ± 1.30 (n = 14)	*p* = 0.276 d = 0.244	18.38 ± 1.16 (n = 28)	18.26 ± 1.32 (n = 14)	18.51 ± 1.00 (n = 14)	*p* = 0.578 d = 0.213	*p* = 0.892 d = 0.027
6 × 6 VD Parafoveal inferior	-	18.39 ± 1.29 (n = 36)	18.55 ± 1.29 (n = 22)	18.14 ± 1.31 (n = 14)	*p* = 0.242 d = 0.314	18.46 ± 1.05 (n = 28)	18.31 ± 1.14 (n = 14)	18.60 ± 0.98 (n = 14)	*p* = 0.482 d = 0.270	*p* = 0.860 d = 0.056
6 × 6 VD Parafoveal temporal	-	18.43 ± 1.20 (n = 36)	18.76 ± 1.08 (n = 22)	17.91 ± 1.23 (n = 14)	*p* < 0.05 * d = 0.732	18.60 ± 0.94 (n = 28)	18.36 ± 1.14 (n = 14)	18.84 ± 0.65 (n = 14)	*p* = 0.192 d = 0.510	*p* = 0.973 d = 0.162
6 × 6 VD Perifoveal superior	-	18.46 ± 1.10 (n = 36)	18.79 ± 0.74 (n = 22)	17.93± 1.36 (n = 14)	*p* < 0.05 * d = 0.788	18.69 ± 1.11 (n = 28)	18.40 ± 1.33 (n = 14)	18.98 ± 0.785 (n = 14)	*p* = 0.357 d = 0.536	*p* = 0.276 d = 0.212
6 × 6 VD Perifoveal nasal	-	19.88± 0.56 (n = 36)	19.97 ± 0.39 (n = 22)	19.72 ± 0.75 (n = 14)	*p* = 0.484 d = 0.429	19.71 ± 1.31 (n = 28)	19.84 ± 0.89 (n = 14)	19.59 ± 1.64 (n = 14)	*p* = 0.800 d = 0.194	*p* = 0.583 d = 0.163
6 × 6 VD Perifoveal inferior	-	18.54 ± 1.20 (n = 36)	18.77 ± 0.98 (n = 22)	18.16 ± 1.44 (n = 14)	*p* = 0.131 d = 0.502	18.51 ± 1.19 (n = 28)	18.54 ± 1.21 (n = 14)	18.49 ± 1.23 (n = 14)	*p* = 0.927 d = 0.035	*p* = 0.903 d = 0.018
6 × 6 VD Perifoveal temporal	-	17.18 ± 1.74 (n = 36)	17.80 ± 1.27 (n = 22)	16.21 ± 1.97 (n = 14)	*p* < 0.01 * d = 0.962	17.64 ± 1.30 (n = 28)	17.27 ± 1.31 (n = 14)	18.00 ± 1.23 (n = 14)	*p* = 0.145 d = 0.567	*p* = 0.375 d = 0.299
6 × 6 PD Central	%	24.55 ± 7.30 (n = 36)	25.44 ± 6.99 (n = 22)	23.16 ± 7.82 (n = 14)	*p* = 0.370 d = 0.306	26.18 ± 5.99 (n = 28)	26.07 ± 6.47 (n = 14)	26.29 ± 5.71 (n = 14)	*p* = 0.765 d = 0.035	*p* = 0.270 d = 0.244
6 × 6 PD Parafoveal complete	%	44.25± 3.09 (n = 36)	44.75 ± 2.90 (n = 22)	43.46 ± 3.34 (n = 14)	*p* = 0.108 d = 0.410	44.00 ± 2.45 (n = 28)	43.48 ± 2.90 (n = 14)	44.53 ± 1.86 (n = 14)	*p* = 0.534 d = 0.431	*p* = 0.330 d = 0.087
6 × 6 PD Perifoveal Complete	%	46.18± 3.06 (n = 36)	47.05 ± 1.89 (n = 22)	44.81 ± 4.01 (n = 14)	*p* < 0.05 * d = 0.711	45.93 ± 2.45 (n = 28)	45.63 ± 2.59 (n = 14)	46.236 ± 2.353 (n = 14)	*p* = 0.550 d = 0.245	*p* = 0.516 d = 0.089
6 × 6 PD Complete	%	45.150 ± 3.045 (n = 36)	45.94 ± 2.14 (n = 22)	43.91 ± 3.86 (n = 14)	*p* < 0.05 * d = 0.648	44.968 ± 2.428 (n = 28)	44.61 ± 2.67 (n = 14)	45.32 ± 2.19 (n = 14)	*p* = 0.629 d = 0.289	*p* = 0.551 d = 0.066
6 × 6 PD Perifoveal superior	%	44.99 ± 2.96 (n = 36)	45.41 ± 2.89 (n = 22)	44.32 ± 3.05 (n = 14)	*p* = 0.194 d = 0.369	44.68 ± 2.77 (n = 28)	43.96 ± 3.42 (n = 14)	45.41 ± 1.76 (n = 14)	*p* = 0.420 d = 0.533	*p* = 0.424 d = 0.108
6 × 6 PD Perifoveal nasal	%	43.51 ± 3.49 (n = 36)	43.75 ± 3.30 (n = 22)	43.13 ± 3.86 (n = 14)	*p* = 0.820 d = 0.174	42.94 ± 2.61 (n = 28)	42.65 ± 3.00 (n = 14)	43.22 ± 2.22 (n = 14)	*p* = 0.572 d = 0.217	*p* = 0.126 d = 0.187
6 × 6 PD Perifoveal inferior	%	44.47 ± 3.29 (n = 36)	44.78 ± 3.24 (n = 22)	43.99 ± 3.45 (n = 14)	*p* = 0.314 d = 0.238	44.26 ± 2.83 (n = 28)	43.86 ± 3.02 (n = 14)	44.66 ± 2.68 (n = 14)	*p* = 0.448 d = 0.283	*p* = 0.556 d = 0.069
6 × 6 PD Perifoveal temporal	%	43.94 ± 3.29 (n = 36)	44.91 ± 2.80 (n = 22)	42.43 ± 3.52 (n = 14)	*p* < 0.01 * d = 0.779	44.15 ± 2.34 (n = 28)	43.51 ± 2.79 (n = 14)	44.79 ± 1.65 (n = 14)	*p* = 0.154 d = 0.558	*p* = 0.761 d = 0.074
6 × 6 PD Parafoveal superior	%	46.61 ± 3.19 (n = 36)	47.61 ± 2.10 (n = 22)	45.09 ± 4.03 (n = 14)	*p* < 0.05 * d = 0.771	46.29 ± 2.66 (n = 28)	45.67 ± 3.05 (n = 14)	46.90 ± 2.15 (n = 14)	*p* = 0.476 d = 0.466	*p* = 0.413 d = 0.110
6 × 6 PD Parafoveal nasal	%	48.89 ± 1.68 (n = 36)	48.98 ± 1.05 (n = 22)	48.74 ± 2.39 (n = 14)	*p* = 0.338 d = 0.133	47.93 ± 2.99 (n = 28)	48.26 ± 1.82 (n = 14)	47.60 ± 3.89 (n = 14)	*p* = 0.982 d = 0.219	*p* = 0.194 d = 0.393
6 × 6 PD Parafoveal inferior	%	46.68 ± 3.69 (n = 36)	47.37 ± 2.55 (n = 22)	45.59 ± 4.90 (n = 14)	*p* = 0.417 d = 0.457	45.68 ± 3.70 (n = 28)	46.16 ± 3.15 (n = 14)	45.20 ± 4.25 (n = 14)	*p* = 0.613 d = 0.256	*p* = 0.109 d = 0.270
6 × 6 PD Parafoveal temporal	%	42.59 ± 4.69 (n = 36)	44.32 ± 3.29 (n = 22)	39.86 ± 5.35 (n = 14)	*p* < 0.01 * d = 1.002	43.01 ± 3.75 (n = 28)	42.42 ± 3.70 (n = 14)	43.60 ± 3.85 (n = 14)	*p* = 0.448 d = 0.312	*p* = 0.823 d = 0.100
FAZ Area	mm^2^	0.228 ± 0.100 (n = 36)	0.219 ± 0.090 (n = 22)	0.242 ± 0.118 (n = 14)	*p* = 0.536 d = 0.209	0.212 ± 0.071 (n = 27)	0.214 ± 0.052 (n = 13)	0.211 ± 0.087 (n = 14)	*p* = 0.911 d = 0.044	*p* = 0.470 d = 0.180
FAZ Perimeter	mm	1.875 ± 0.499 (n = 36)	1.850 ± 0.478 (n = 22)	1.914 ± 0.546 (n = 14)	*p* = 0.712 d = 0.125	1.850 ± 0.323 (n = 27)	1.889 ± 0.284 (n = 13)	1.814 ± 0.363 (n = 14)	*p* = 0.558 d = 0.230	*p* = 0.819 d = 0.060
FAZ Circularity	-	0.773 ± 0.076 (n = 36)	0.774 ± 0.070 (n = 22)	0.771 ± 0.087 (n = 14)	*p* = 0.909 d = 0.043	0.748 ± 0.090 (n = 27)	0.753 ± 0.073 (n = 13)	0.7423± 0.106 (n = 14)	*p* = 0.922 d = 0.112	*p* = 0.179 d = 0.300

## 4. Discussion

This study directly addresses a persistent gap in the pediatric OCT and OCT-A literature by delivering device-consistent, within-study comparisons between pediatric and adult cohorts. Many prior reports either focus solely on pediatric normative values or perform indirect comparisons against historical adult databases, a practice that introduces device heterogeneity, temporal protocol drift and population sampling biases. Our unified acquisition and processing protocol removes those confounders and therefore allows a more reliable attribution of observed differences to true biological age effects rather than methodological artifacts [6,7].

### 4.1. Direct Pediatric–Adult Comparisons

#### 4.1.1. RNFL Thickness and Distribution Patterns

Direct pediatric–adult comparison showed no significant differences in global RNFL thickness or quadrant measurements. This aligns with indirect reports that pediatric RNFL values generally fall within adult reference ranges [8,9]. The classical I > S > N > T topography was preserved in both groups, and despite documented quadrant variability in the literature [10], our cross-age consistency supports the use of population- and device-specific normative databases and suggests the ISNT rule remains clinically useful when applied with appropriate, age-matched references. Our findings of stable RNFL thickness across pediatric age groups are consistent with the foundational handheld OCT work by Patel et al., who demonstrated reliable optic nerve head measurements in infants and children using portable systems [5]. The preservation of the ISNT pattern across all ages in our tabletop OCT study validates earlier observations from handheld studies, while providing enhanced precision through improved signal strength and reduced motion artifacts.

#### 4.1.2. Macular Thickness and Distribution Patterns

Adults showed significantly thicker total MT than pediatric subjects (270.36 ± 17.02 μm vs. 251.67 ± 21.32 μm; *p* < 0.001), an average difference of 18.69 μm that was driven by all parafoveal sectors while perifoveal measurements remained comparable. This age-related divergence echoes prior indirect findings and indicates that parafoveal changes, rather than central foveal or perifoveal shifts, account for most developmental differences in macular architecture [8]. The developmental changes we observed align with the comprehensive framework established by Toth and colleagues, who emphasized the critical relationship between retinal development and brain maturation [4]. Our quantified difference in macular thickness between children and adults provides tabletop OCT validation of developmental patterns first identified through handheld imaging systems.

Lever et al. provide strong external validation: their cross-sectional study of 157 healthy subjects aged 10–79, using Heidelberg SPECTRALIS SD-OCT, demonstrated a significant age-related decline in whole macular thickness of approximately 0.13 µm per year, confirmed by one-way ANOVA and linear regression [11]. Crucially, these morphological changes were localized to the parafoveal and perifoveal ETDRS subfields while the central foveal C0 subfield remained unaffected, directly aligning with our finding of significant parafoveal differences between children and adults and no change in central foveal parameters—supporting the view that observed parafoveal thinning reflects true developmental change rather than measurement artifacts.

Apparent quadrant-order differences between pediatric (S > N > I > T) and adult (N > S > I > T) groups are numerically negligible—the superior and nasal parafoveal values in children differ by only 0.02 μm, well within OCT measurement variability—so the practical distribution is effectively equivalent across ages. The temporal sector remained the thinnest region throughout, a pattern consistent with pediatric normative data [12], while perifoveal N > S > I > T ordering was identical in both cohorts with only minimal absolute differences.

#### 4.1.3. GCIPL Analysis and Regional Patterns

GCIPL analysis showed no significant thickness differences between children and adults across measured sectors, but the regional distribution differed: children followed an NI > TI > NS > S > I > TS order, whereas adults showed S > NS > TI > TS > NI > I. Although absolute sectoral differences were not statistically significant, the shift from inferior-dominant to superior-dominant patterns implies that ganglion cell complex maturation is regionally uneven, so pediatric interpretation should account for age-specific distribution rather than rely on absolute values alone. These regional pattern differences likely reflect the protracted postnatal development of inner retinal layers, where different anatomical sectors mature at varying rates during childhood and adolescence. Retinal development follows established central-to-peripheral and temporal-to-nasal gradients, with ganglion cell layer organization and synaptic connectivity continuing to evolve throughout the pediatric period. The observed shift from inferior-dominant to superior-dominant GCIPL patterns represents the natural progression toward adult retinal architecture, emphasizing that pediatric interpretation should account for these developmental topographical variations rather than relying solely on absolute thickness values.

This finding contrasts with Lever et al., who reported modest age-related GC layer declines (0.022–0.10 µm/year) across ages 10–79 using SPECTRALIS [11]; the discrepancy likely reflects platform segmentation differences (we used Zeiss Cirrus’ combined GCIPL metric, whereas Lever analyzed layers separately) and their inclusion of older adults where degenerative loss is more apparent. Pua et al.’s observation—that GCIPL aligns better between pediatric and adult databases than macular thickness—supports our conclusion that regional patterning, rather than mean thickness, is most relevant for pediatric GCIPL assessment. Pua et al.’s observation—that GCIPL aligns better between pediatric and adult databases than macular thickness [8]—supports our conclusion that regional patterning, rather than mean thickness, is most relevant for pediatric GCIPL assessment.

#### 4.1.4. OCT-A Parameters

Children demonstrated significantly higher PF values than adults in multiple regions, while FI did not differ overall. No prior studies were found directly comparing ONH parameters between pediatric and adult populations for benchmarking. ONH PF followed a consistent temporal > inferior > superior > nasal pattern in both groups, with the temporal quadrant highest (children 47.81%, adults 46.54%), indicating that the gross vascular architecture and regional perfusion hierarchy are preserved through development despite absolute VD differences.

FI revealed subtler, age-dependent redistributions: children exhibited a temporal > nasal > superior > inferior pattern, whereas adults showed temporal > nasal > inferior > superior, with the temporal quadrant remaining dominant in both. This divergence implies that total vessel area (perfusion) maintains a stable regional order across ages, while capillary perfusion intensity shifts modestly during maturation—notably a superior>inferior FI in children that may reverse in adults. Clinically, these patterns establish age-appropriate expectations for ONH vascular assessment: anticipate temporal dominance for both PF and FI, with superior–inferior FI rankings varying by age, and an overall PF hierarchy of temporal > inferior > superior > nasal across all ages.

For macular parameters, no significant differences were observed between children and adults across all measured parameters, suggesting that macular microvascular architecture reaches adult-equivalent maturity earlier in development compared to ONH vasculature. Our OCT-A findings present an interesting contrast to the age-related vascular changes reported by Lever et al., who demonstrated significant decreases in superficial capillary plexus VD with advancing age (36.4% in youngest group to 26.1% in oldest group) [11]. While we found no significant differences in macular VD parameters between children and adults, we did observe significant age-related differences within our pediatric population, with younger children consistently demonstrating higher vessel and perfusion density values compared to older children.

For macular parameters, no significant differences were observed between children and adults across all measured metrics, implying that the macular microvascular architecture attains adult-equivalent maturity earlier in development than the optic nerve head vasculature. Although macular VD did not differ between pediatric and adult cohorts, we observed clear age-related changes within the pediatric group: younger children (1–9 years) consistently showed higher vessel and perfusion density values than older children (10–17 years). This pattern indicates that the principal phase of macular vascular remodeling occurs during childhood rather than across the childhood–adulthood transition.

Our OCT-A results contrast with Lever et al., who reported a marked decline in superficial capillary plexus VD across ages (from 36.4% in the youngest to 26.1% in the oldest groups) [11]; this apparent discrepancy likely reflects distinct developmental phases and differences in cohort age ranges and device methodology. Indirect pediatric comparisons support early macular stability: Bajtl et al. found mean FAZ ≈ 0.28 mm^2^ in four-year-olds [13] and Comba et al. reported FAZ 0.25 ± 0.10 mm^2^ in Turkish children, both lying within adult literature ranges [14]. Lever et al. likewise reported a consistent FAZ across their age span, and our study found no pediatric–adult FAZ differences [8]. Together, these concordant observations endorse FAZ as an age-independent biomarker and substantiate the conclusion that macular microvascular maturation is largely completed in early childhood.

### 4.2. Pediatric Subgroup Analysis

#### 4.2.1. RNFL Parameters

RNFL thickness was significantly thinner only at the 5 and 6 o’clock clock-hour positions in the younger children, with no significant differences at the quadrant level. These two isolated findings at single clock positions are likely not clinically relevant, supporting the overall stability of RNFL measurements across pediatric age groups.

#### 4.2.2. Macular Analysis

Across pediatric subgroups we found no significant differences in MT or GCIPL measurements; OCT parameters remained stable throughout childhood. This stability is corroborated by Jammal et al., who divided 144 Jordanian children into three age bands (<10, 10–12, >12 years) and reported no significant variation in macular thickness or volume across ETDRS sectors [15]. The concordance between independent cohorts using the same OCT platform (German versus Jordanian children) reinforces the conclusion that macular structural metrics are largely invariant across pediatric age groups and therefore reliable for clinical and research use without extensive age-adjustment within childhood.

#### 4.2.3. OCT-A Vascular Patterns

Within the pediatric cohort we observed consistent age-related differences in vascular metrics: younger children exhibited higher PF in ONH regions—most notably in the complete outer, superior and inferior zones—indicating ongoing ONH vascular maturation during childhood. Similarly, macular VD and PD were higher in younger (1–9 years) versus older (10–17 years) children across both 3 × 3 mm and 6 × 6 mm scans, with the parafoveal and temporal sectors showing the greatest divergence. Despite these peripheral and parafoveal changes. FAZ measurements remained stable, implying that central foveal vasculature attains its mature state early while peripheral macular networks continue to remodel through the first decade.

Kurumoğlu et al., who analyzed 370 eyes across four pediatric age groups (7–18 years), reported minimal ONH vascular changes and no significant differences in superficial capillary plexus VD or FAZ between groups, which diverges from our intra-pediatric vascular findings [16]. This discrepancy likely stems from platform differences—we used Zeiss CIRRUS while Kurumoğlu employed AngioVue—each with distinct segmentation algorithms and measurement methodologies that affect absolute and regional VD metrics. Thus, cross-study comparisons must account for device-specific biases; nonetheless, the recurring theme across studies is FAZ stability and the need to interpret peripheral macular OCT-A changes within the context of developmental stage and imaging platform.

#### 4.2.4. Impact of Biometric Differences

The significant difference in axial length between children and adults (22.90 ± 0.99 mm vs. 23.84 ± 0.84 mm; *p* < 0.01) was expected, as it is well established that axial length increases significantly during the first few years of life as part of normal ocular development. While this difference warrants consideration of potential magnification effects on our measurements, as demonstrated by Savini et al. who showed that RNFL thickness and optic disk parameters can be influenced by axial length variations [17], several factors support the validity of our age-related findings: First, the magnitude of macular thickness differences we observed substantially exceeds the expected magnification-related measurement variations. Second, RNFL thickness measurements, which Savini et al. demonstrated are more susceptible to axial length effects, showed no significant differences between groups, arguing against systematic magnification bias. Third, our OCT-A findings of higher ONH perfusion in children are consistent with known developmental vascular patterns and unlikely to be artifacts of ocular magnification. Nevertheless, future studies incorporating axial length correction algorithms may provide additional refinement of age-specific normative values.

### 4.3. Clinical Implementation and Technical Feasibility

The substantial differences between children and adults, together with distinct regional shifts in GCIPL and MT, make clear that age-specific reference ranges are required rather than extrapolating from adult norms. Using adult reference values risks misclassifying normal pediatric development as pathology or overlooking early disease in children and should therefore be avoided. Reporting algorithms, automated flags and clinical thresholds must be stratified by meaningful pediatric age bands to ensure accurate referrals and management.

The documented distribution patterns provide clinicians with age-appropriate expectations for regional thickness hierarchies, enabling more accurate interpretation of pediatric OCT measurements. For example, children typically show inferior-dominant GCIPL patterns while adults demonstrate superior-dominant patterns, and ONH perfusion shows temporal dominance across all ages with age-related inferior-superior flow index reversals.

High-quality OCT and OCT-A acquisition in children is practicable with modern devices and tailored acquisition protocols; this supports routine implementation and the development of pediatric-specific interpretation guidelines. Integrating age-stratified normative databases into device software, standardizing pediatric protocols and training staff will enable reliable imaging, reduce unnecessary follow-ups and ensure imaging meaningfully informs pediatric clinical care. The technical success demonstrated in our study builds directly upon the pioneering protocols established by Maldonado et al. [3]. While their handheld approach overcame initial barriers to pediatric imaging, our 100% acquisition success rate with tabletop systems demonstrates the maturation of pediatric OCT from specialized research tools to routine clinical instruments. This evolution, supported by the comprehensive developmental framework outlined by Toth [4] and normative databases from groups like Gottlob’s Leicester team [5], positions OCT as an essential component of modern pediatric ophthalmology practice.

### 4.4. Study Limitations

Our cohort of 65 participants, while sufficient for primary group comparisons, offers limited statistical power for detecting subtle subgroup effects, notably gender-specific differences. While sex distribution differed between groups (children 56.8% female vs. adults 82.1% female, *p* = 0.058), this imbalance is unlikely to affect our conclusions, as established literature consistently demonstrates that sex-related differences in RNFL thickness, macular thickness, and retinal vascular density are minimal. The cross-sectional design prevents tracking individual trajectories, so we cannot confirm true longitudinal maturation or distinguish transient variations from consistent developmental trends; prospective follow-up would be required to robustly characterize age-related changes in retinal structure and vasculature.

The study population comprised exclusively healthy Caucasian individuals from Central Europe, which constrains external validity: established ethnic variations in retinal metrics mean our normative values may not apply to non-Caucasian groups or to populations in other geographic regions. Furthermore, data were acquired on a single OCT-A platform, and inter-device variability in segmentation and quantification is well documented, so absolute values may differ when measured on alternative systems

Age distribution within the pediatric cohort was uneven, with relatively few subjects in the 1–5 years range, risking omission of early critical developmental changes. The broad pediatric span (approximately 1–17 years) covers multiple biological stages; grouping across this range may mask age-specific effects that narrower, more granular age bands would reveal.

## 5. Conclusions

Our direct comparison of pediatric and adult cohorts demonstrates significant age-related differences in retinal metrics that caution strongly against extrapolating adult normative data to children. Although RNFL thickness was largely stable across age groups, we observed notable differences in macular thickness, distinct regional shifts in GCIPL distribution, and substantial variation in vascular parameters; collectively, these findings mandate age-specific reference ranges for accurate pediatric interpretation. Relying on adult norms risks both over- and under-diagnosis, with attendant consequences for referral, follow-up and treatment decisions.

The documented regional distribution patterns furnish clinicians with concrete, age-appropriate frameworks for interpreting pediatric OCT and OCT-A scans, while the intra-pediatric vascular differences underscore the necessity of considering developmental stage when assessing microvascular metrics. Achieving consistent, high-quality imaging across the pediatric age spectrum confirms the technical feasibility of routine OCT and OCT-A in clinical practice and supports the incorporation of pediatric-specific thresholds into reporting software and clinical pathways.

These results constitute a pivotal step towards optimizing advanced retinal imaging for children: by adopting age-stratified norms and region-focused templates, clinicians can enhance diagnostic accuracy, improve longitudinal monitoring and ensure imaging contributes meaningfully to pediatric ophthalmic care.

## Data Availability

The original contributions presented in this study are included in the article. Further inquiries can be directed to the corresponding author.

## Abbreviations

C/D	Cup-to-disk ratio
ETDRS	Early Treatment Diabetic Retinopathy Study
FAZ	Foveal Avascular Zone
FI	Flow Index
GCIPL	Ganglion Cell-Inner Plexiform Layer
I	Inferior
ILM	Inner Limiting Membrane
IPL	Inner Plexiform Layer
ISNT	Inferior > Superior > Nasal > Temporal
LogMAR	Logarithm of Minimum Angle of Resolution
mm	Millimeter
mm^2^	Square Millimeter
MT	Macular Thickness
μm	Micrometer
N	Nasal
NI	Nasal Inferior
NS	Nasal Superior
OCT	Optical Coherence Tomography
OCT-A	Optical Coherence Tomography Angiography
ONH	Optic Nerve Head
PD	Perfusion Density
PF	Perfusion
RNFL	Retinal Nerve Fiber Layer
RNFLT	Retinal Nerve Fiber Layer Thickness
RPC	Radial Peripapillary Capillary
RPE	Retinal Pigment Epithelium
S	Superior
SD	Standard Deviation
SD-OCT	Spectral Domain Optical Coherence Tomography
T	Temporal
TI	Temporal Inferior
TS	Temporal Superior
VD	Vessel Density
%	Percent
